# Host-Cell-Dependent Roles of E-Cadherin in *Serratia* Invasion

**DOI:** 10.3390/ijms242317075

**Published:** 2023-12-03

**Authors:** Olga Tsaplina, Ekaterina Lomert, Yuliya Berson

**Affiliations:** Institute of Cytology, Russian Academy of Sciences, Tikhoretsky av. 4, 194064 St Petersburg, Russia; e.lomert@gmail.com (E.L.); juletschka.ber@gmail.com (Y.B.)

**Keywords:** bacterial invasion, E-cadherin, ADAM10, hemolysin ShlA

## Abstract

Bacteria use cell surface proteins to mediate host–pathogen interactions. Proteins responsible for cell adhesion, including E-cadherin, serve as receptors for entry into the host cell. We have previously shown that an increase in eukaryotic cell sensitivity to *Serratia grimesii* correlates with an increase in E-cadherin expression. On the other hand, *Serratia proteamaculans* invasion involves the EGFR, which can interact with E-cadherin on the surface of host cells. Therefore, we investigated the role of E-cadherin in *Serratia* invasion into M-HeLa and Caco-2 cells. Bacterial infection increased E-cadherin expression in both cell lines. Moreover, E-cadherin was detected in the Caco-2 cells in a full-length form and in the M-HeLa cells in only a truncated form in response to incubation with bacteria. Transfection with siRNA targeting E-cadherin inhibited *S. proteamaculans* invasion only into the Caco-2 cells. Thus, only full-length E-cadherin is involved in *S. proteamaculans* invasion. On the other hand, transfection with siRNA targeting E-cadherin inhibited *S. grimesii* invasion into both cell lines. Thus, not only may full-length E-cadherin but also truncated E-cadherin be involved in *S. grimesii* invasion. Truncated E-cadherin can be formed as a result of cleavage by bacterial proteases or the Ca^2+^-activated cellular protease ADAM10. The rate of Ca^2+^ accumulation in the host cells depends on the number of bacteria per cell upon infection. During incubation, Ca^2+^ accumulates only when more than 500 *S. grimesii* bacteria are infected per eukaryotic cell, and only under these conditions does the ADAM10 inhibitor reduce the sensitivity of the cells to bacteria. An EGFR inhibitor has the same quantitative effect on *S. grimesii* invasion. Apparently, as a result of infection with *S. grimesii*, Ca^2+^ accumulates in the host cells and may activate the ADAM10 sheddase, which can promote invasion by cleaving E-cadherin and, as a result, triggering EGFR signaling. Thus, the invasion of *S. proteamaculans* can only be promoted by full-length E-cadherin, and *S. grimesii* invasion can be promoted by both full-length and truncated E-cadherin.

## 1. Introduction

Cadherins form a large group of transmembrane receptors that mediate specific intercellular adhesion. All cadherins have extracellular domains that are usually organized in tandem repeats that mediate Ca^2+^-dependent homophilic interactions [1]. The extracellular domain of E-cadherin (epithelial cadherin) is involved in the Ca^2+^-dependent intercellular homotypic interactions required for the formation of adherens junctions, whereas its cytoplasmic domain interacts with the cell actin cytoskeleton via α- and β-catenins that stabilize cell junctions [2].

In addition to their adhesive function, cadherins mediate host–pathogen interactions. These cell surface proteins are used by a variety of pathogens during host cell entry to mediate host–pathogen interactions. The best-studied bacterial surface protein internalin A (InlA) of *Listeria monocytogenes* mediates the infection of nonphagocytic cells by facilitating bacterial adhesion through specific interaction with E-cadherin [3]. The interaction of InlA with E-cadherin activates the downstream signaling pathways that trigger the endocytosis of the pathogen [4]. In addition, while some virulence factors adhere and bind to cadherins to get endocytosed, other bacterial proteins promote infection by functioning as proteases that degrade cadherins and facilitate bacterial entry into the host cell [5]. At present, the physiological functions mediated by cadherins are well established, although the study of their roles as mediators of host–pathogen interactions is still in its nascent stages.

It was previously shown that the treatment of eukaryotic cells with the antioxidants N-acetylcysteine and dihydrolipoic acid, as well as with a selective inhibitor of Rho-associated protein kinase (ROCK), not only promotes the expression of E-cadherin but also increases the sensitivity of M-HeLa cells to invasion by the bacteria *Serratia grimesii* [6,7,8]. This allowed us to suggest that E-cadherin is involved in *S. grimesii* invasion. It was also shown that invasion of the bacteria *Serratia proteamaculans* involves the epidermal growth factor receptor (EGFR), which can interact with E-cadherin on the surface of eukaryotic cells [9,10]. Therefore, the aim of this work was to evaluate and compare the role of E-cadherin in *S. grimesii* and *S. proteamaculans* invasion.

Bacteria of the *Serratia* genus are known to be facultative pathogens able to induce nosocomial infections or infections in immunocompromised patients [11]. They are found in patients with pneumonia and infections of the urinary tract, wound infections and circulatory infections. In addition, *Serratia* can colonize the intestinal mucosa [11]. Therefore, in this work, we determined the mechanism used by *Serratia* to penetrate into the epithelial-like cells of cervical carcinoma M-HeLa and colorectal adenocarcinoma Caco-2 cell lines in vitro. In this work, we showed that infection with *Serratia* leads to an increase in E-cadherin expression and assessed the redistribution of the receptor in response to bacterial infection. After incubation with bacteria, E-cadherin remains full-length in Caco-2 cells and truncated in M-HeLa cells. E-cadherin can accumulate in a truncated form as a result of cleavage by bacterial proteases or Ca^2+^-activated disintegrin and metalloproteinase 10 (ADAM10). We showed that only *S. grimesii* infection leads to the accumulation of Ca^2+^ in the host cell, and their invasion is inhibited by a selective inhibitors of the host cell proteins, namely, ADAM10 and EGFR. Apparently, as a result of infection with *S. grimesii*, Ca^2+^ accumulates in the host cells and may activate the ADAM10 sheddase, which can promote invasion by cleaving E-cadherin and, as a result, triggering EGFR signaling. Moreover, we showed that E-cadherin is involved in the invasion of *S. proteamaculans* only into Caco-2 cells but not into M-HeLa cells. On the other hand, E-cadherin is involved in the invasion of *S. grimesii* into cells of both cell lines. Thus, the invasion of *S. proteamaculans* can be promoted only by full-length E-cadherin, and the invasion of *S. grimesii* can be promoted by both full-length and truncated E-cadherin.

## 2. Results

**Figure 1:**
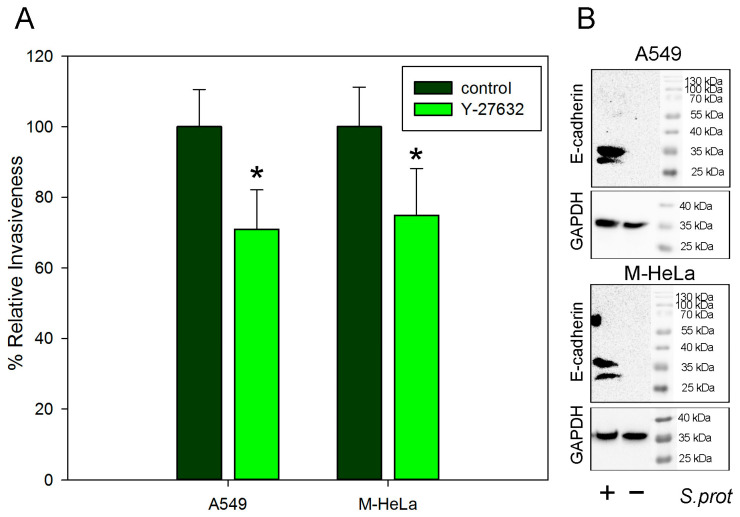
(**A**) The effect of pre-incubation of A549 and M-HeLa cells with ROCK inhibitor (10 µM Y-27632) for 30 min at 37 °C on the invasion intensity of *S. proteamaculans* after 48 h of growth. Control—intensity of invasion into untreated cells. The number of intracellular bacteria was estimated as a percentage, taking the number of intracellular bacteria in control samples as 100%. Values are expressed as mean S.D. (error bars). A difference to the control was considered significant at the * *p* < 0.05 level. (**B**) Total E-cadherin and internal control glyceraldehyde 3-phosphate dehydrogenase (GAPDH) in A549 and M-HeLa cells before and after 2 h of incubation with *S. proteamaculans*.

We have previously shown that treatment of eukaryotic cells with a ROCK inhibitor results in a 2–3-fold increase in the sensitivity of these cells to *S. grimesii* invasion [8]. This effect was correlated with a four-fold increase in E-cadherin expression [8]. Therefore, we compared how treatment with a ROCK inhibitor affects the sensitivity of cells of different origins to *S. proteamaculans*. Specifically, we quantified the effect of a ROCK inhibitor on the sensitivity to bacteria of cervical carcinoma M-HeLa cells, which normally do not synthesize E-cadherin [12], and adenocarcinoma alveolar basal epithelial A549 cells, which should synthesize the E-cadherin characteristic of epithelial cells [2]. Pre-incubation with a ROCK inhibitor reduced the sensitivity of the human M-HeLa cervical adenocarcinoma cells and adenocarcinoma A549 alveolar basal epithelial cells to bacteria by 20–25% (Figure 1A). The decrease in cell sensitivity in response to the inhibitor treatment did not depend on the origin of the cell lines. Thus, an increase in E-cadherin expression in response to the treatment with the Y-27632 inhibitor did not lead to an increase in *S. proteamaculans* invasion. Based on our results, we hypothesized that the contribution of E-cadherin to *S. proteamaculans* invasion is smaller than that to *S. grimesii* invasion. Therefore, we compared the participation of E-cadherin in the invasion of *S. grimesii* and *S. proteamaculans*.

In addition, the effect of *S. proteamaculans* infection on the accumulation of E-cadherin in host cells was evaluated by Western blot analysis (Figure 1B). Figure 1B shows that after co-incubation with *S. proteamaculans*, E-cadherin fragments of about 33 and 38 kDa were detected in the cells. Before infection, both cell lines did not synthesize E-cadherin in amounts sufficient for detection. These results confirm the data from confocal microscopy. It is probable that a small amount of E-cadherin accumulated due to the fact that the cells in the experiment did not form a 100% monolayer rich in intercellular contacts. Therefore, in further work, we chose colorectal adenocarcinoma Caco-2 cells as epithelial cells synthesizing E-cadherin. 

**Figure 2:**
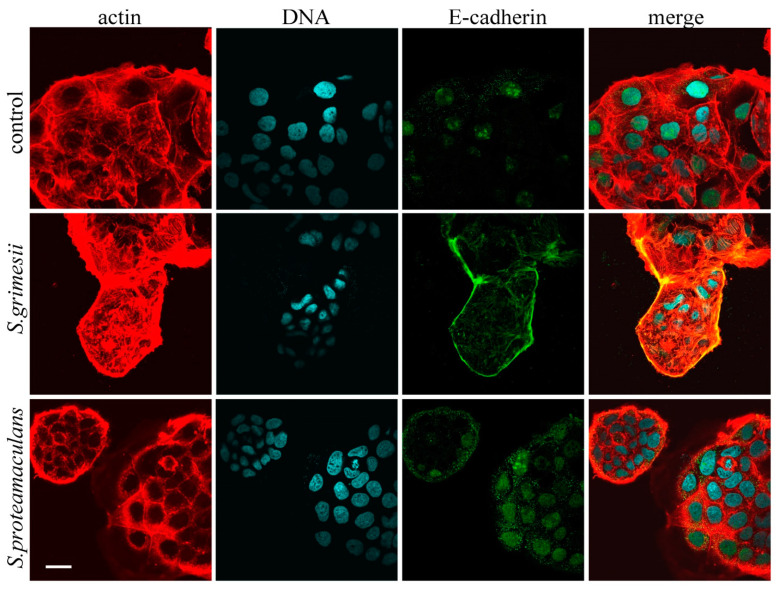
Distribution of E-cadherin in Caco-2 cells as a result of *S. grimesii* and *S. proteamaculans* invasion. Cells were incubated with bacteria for 2 h after bacteria sedimentation by centrifugation. Control—uninfected Caco-2 cells. Cytoskeleton was stained with rhodamine-phalloidin; E-cadherin was stained with antibodies; DNA was stained with DAPI. Scale bar: 30 μm.

**Figure 3:**
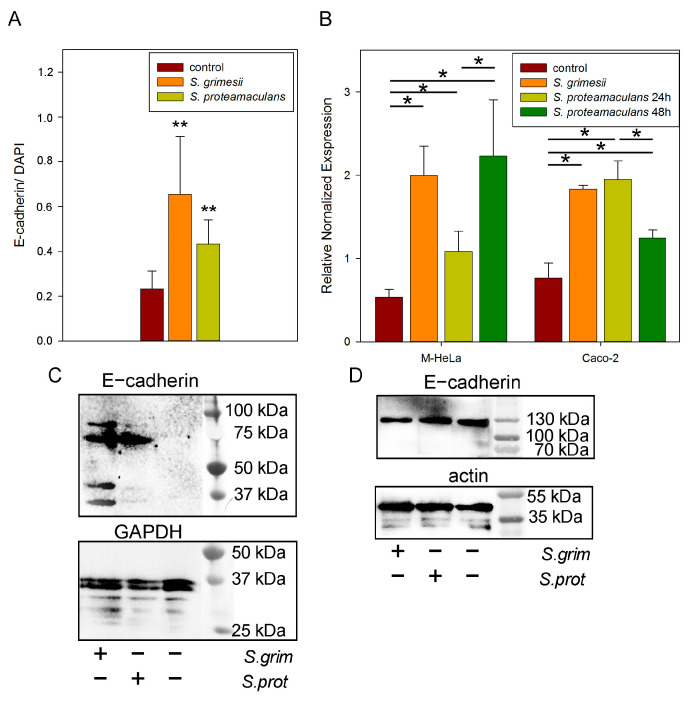
E-cadherin in *Serratia* invasion. (**A**). Effect of bacterial infection on E-cadherin in the host cell. The mean fluorescence intensity of E-cadherin normalized to the mean fluorescence intensity of DAPI estimated with ImageJ on 6–9 images obtained using confocal microscopy (Figure 2 shows an example). Values are expressed as mean S.D. (error bars). A difference to the control was considered significant at the ** *p* < 0.01 level. (**B**). Effect of bacterial infection on E-cadherin expression in the host cell. Using real-time RT-PCR, E-cadherin expression was determined after 2 h of incubation with *S. grimesii* and *S. proteamaculans* after 24 and 48 h of growth. Control—uninfected M-HeLa and Caco-2 cells. GADPH and β -actin served as an internal control. Values are expressed as mean S.D. (error bars). A difference was considered significant at the * *p* < 0.05 level. (**C**). The total amount of E-cadherin and internal control GAPDH in M-HeLa cells before and after infection with *S. grimesii* and *S. proteamaculans*. Before the experiment, bacteria were grown for 48 h. (**D**). The total amount of E-cadherin and internal control actin in Caco-2 cells before and after infection with *S. grimesii* and *S. proteamaculans*. Before the experiment, bacteria were grown for 24 h.

We assessed the distribution of E-cadherin in response to incubation of cells with bacteria. The amount of E-cadherin in the M-HeLa cells did not allow us to assess the distribution of this receptor using immunofluorescent microscopy. Therefore, we used Caco-2 colorectal adenocarcinoma cells to assess the E-cadherin redistribution (Figure 2). In the control cells, E-cadherin was localized in the nuclei and in the cytoplasmic granules (Figure 2), which are endosomes [13]. After incubation with *S. proteamaculans*, the number and size of endosomes stained with antibodies to E-cadherin increased. When the cells were infected with *S. grimesii*, E-cadherin accumulated along the cell perimeter. Thus, according to the confocal microscopy results, infection by each of these bacterial strains led to the accumulation of E-cadherin (Figure 3A), but the receptor was redistributed in different compartments of the host cell (Figure 2). 

We have previously shown that infection with *S. proteamaculans* leads to an increase in the host cells’ expression of β1 integrin and EGF receptor involved in invasion [10]. Using real-time RT-PCR, here, we showed that infection with both bacteria strains increased the E-cadherin expression in the Caco-2 and M-Hela cells by 2.5 and 4 times, respectively (Figure 3B). At the same time, infection with *S. proteamaculans* at the early stationary phase of growth (24 h) of the HeLa cells and at the late stationary phase of growth (48 h) of the Caco-2 cells had a weaker effect on E-cadherin expression. Western blot analysis confirmed the accumulation of E-cadherin in response to bacterial infection only in the M-HeLa cells (Figure 3C,D). E-cadherin was only detected in the M-HeLa cells infected with *Serratia*. In the present experiment, E-cadherin accumulated not only in the fragments of 33 and 38 kDa but also with the formation of additional two fragments with a molecular weight of about 70 and 80 kDa. The 38 and 33 kDa fragments were found in the cells in smaller amounts after incubation with *S. proteamaculans* than after incubation with *S. grimesii*.

**Figure 4:**
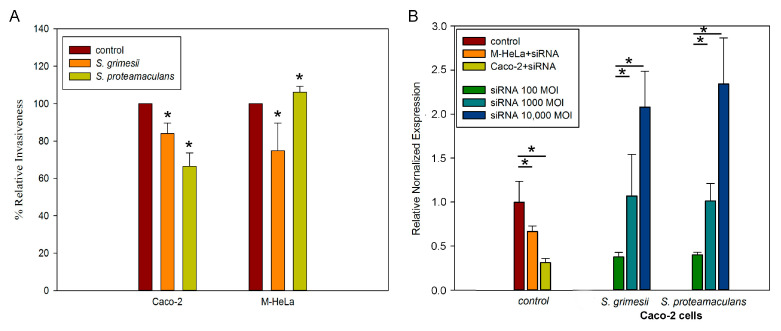
Effect of transfection of eukaryotic cells with siRNA targeting E-cadherin on cell sensitivity to *S. grimesii* and *S. proteamaculans* invasion. (**A**). Effect of transfection of M-HeLa and Caco-2 cells with siRNA targeting E-cadherin on cell sensitivity to *S. grimesii* and *S. proteamaculans* invasion. Before the experiment, bacteria were grown for 24 h. Control—intensity of invasion into cells transfected with siRNA containing scrambled nucleotide sequence. The number of intracellular bacteria was estimated as a percentage, taking the number of intracellular bacteria in control samples as 100%. Values are expressed as mean S.D. (error bars). A difference to the control was considered significant at the * *p* < 0.05 level. (**B**). Effect of bacterial infection on E-cadherin expression in the host cell transfected with siRNA targeting E-cadherin. Using real-time RT-PCR, E-cadherin expression was determined after 2 h of incubation with *S. grimesii* and *S. proteamaculans* after 24 h of growth. Control—uninfected cells transfected with siRNA containing scrambled nucleotide sequence. GADPH and β -actin served as an internal control. Values are expressed as mean S.D. (error bars). A difference was considered significant at the * *p* < 0.05 level.

To confirm the involvement of E-cadherin in *Serratia* invasion, we used siRNA targeting E-cadherin. Transfection of the Caco-2 cells with siRNA reduced the intensity of the invasion of *S. proteamaculans* by 35% and of *S. grimesii* by 15% (Figure 4A). Thus, E-cadherin is involved in the invasion of *Serratia* into Caco-2 cells. On the other hand, transfection of the M-HeLa cells with siRNA reduced the intensity of the invasion of *S. grimesii* by 25% and increased the intensity of the invasion of *S. proteamaculans* by 5% (Figure 4A). Using real-time RT-PCR, we showed that transfection of the M-HeLa cells with siRNA targeting E-cadherin reduced the intensity of E-cadherin expression by 70% in the Caco-2 cells and by 35% in the M-HeLa cells (Figure 4B). On the other hand, after transfection of the cells with siRNA targeting E-cadherin, the growth medium was changed for 1–3 days, and then bacteria causing E-cadherin accumulation were added. Thus, the addition of bacteria may partially neutralize the transfection of cells with siRNA. According to the RT-PCR data, the addition of 1000 bacteria per cell to the siRNA-transfected cells increased the expression of E-cadherin to the level of expression in the control untransfected cells, and the addition of 10,000 bacteria per cell increased the expression of E-cadherin an additional 2-fold (Figure 4B).

**Figure 5:**
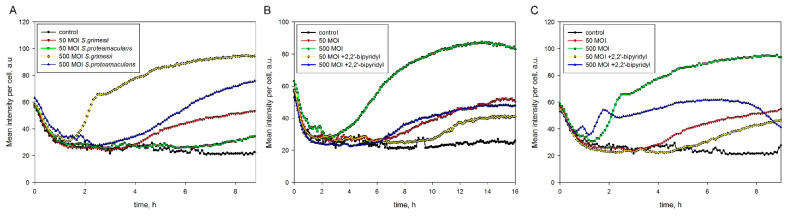
Effect of bacterial infection on Ca^2+^ accumulation in the host cells. Using a CQ1 confocal image cytometer Yokogawa, the mean intensity of Fluo-4-stained Ca^2+^ in the cell was quantified. Effect of infection with *S. grimesii* and *S. proteamaculans* at an MOI of 50 or 500 (**A**). Effect of infection with *S. proteamaculans* (**B**) and *S. grimesii* (**C**) growing in the absence or presence of 0.3 mM 2,2′-bipyridyl at an MOI of 50 or 500. Before the experiment, *S. grimesii* and *S. proteamaculans* were grown for 24 and 48 h, respectively. Control—uninfected M-HeLa cells.

E-cadherin in the M-HeLa cells accumulated in a cleaved form with the formation of fragments of about 80, 70, 38, and 33 kDa. Bacteria can induce E-cadherin cleavage both directly by bacterial proteases and by activating the host cell transmembrane metalloprotease ADAM10, which mediates the regulated shedding of the E-cadherin molecule in fibroblasts and keratinocytes [14]. ADAM10 can be activated by Ca^2+^ influx through host cell pores formed by pore-forming bacterial toxins, including the ShlA toxin from *Serratia marcescens* [15]. Therefore, we quantified Ca^2+^ accumulation in the eukaryotic cells at a multiplicity of infection (MOI) of 50 and 500 bacteria per cell. Our analysis showed that Ca^2+^ accumulated in cells during the two hours of incubation only when the infection intensity of *S. grimesii* increased to 500 bacteria per cell (Figure 5A). Ca^2+^ accumulated more intensively in response to *S. grimesii* infection compared to *S. proteamaculans* infection. It is possible that this was due to the fact that the bacteria *S. grimesii* divide during incubation, while the bacteria *S. proteamaculans* grow at a lower temperature and do not divide at 37 °C.

Previously, we have shown that only *S. proteamaculans*, but not *S. grimesii*, synthesizes the active ShlA toxin [16]. To quantify the toxin’s contribution to Ca^2+^ accumulation, we activated the ShlA toxin of *S. proteamaculans* using 2,2’-bipyridyl. The hemolytic activity of ShlA is dependent on the availability of iron [17], and iron limitation during bacterial growth in a medium with 2,2’-bipyridyl, which is an iron chelator, causes an increase in the hemolytic activity of ShlA [16,18]. However, infection with *S. proteamaculans* growing in an iron-depleted medium slowed rather than accelerated the accumulation of Ca^2+^ in the infected M-HeLa cells (Figure 5B). To test whether this effect was due to activation of the toxin ShlA, we evaluated the effect of 2,2’-bipyridyl on *S. grimesii*, which does not produce the active toxin ShlA [16]. Infection with *S. grimesii* growing in an iron-depleted medium also slowed down the accumulation of Ca^2+^ in the M-HeLa cells (Figure 5C). Thus, infection with *S. grimesii* and *S. proteamaculans* leads to the accumulation of Ca^2+^ in host cells by a mechanism independent of the pore-forming toxin ShlA.

**Figure 6:**
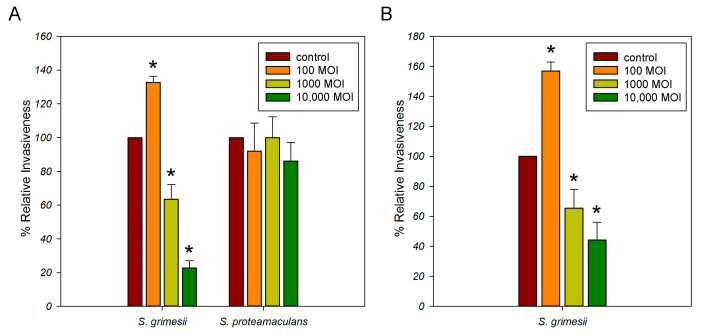
The effect of pre-incubation of M-HeLa cells with inhibitors on the invasion intensity of *Serratia.* (**A**). The effect of pre-incubation of M-HeLa cells with inhibitor of ADAM10 metalloprotease (20 µM GI 254023X) for 30 min on the invasion intensity of *S. grimesii* and *S. proteamaculans* after 24 h of growth. Control—intensity of invasion into untreated cells. The number of intracellular bacteria was estimated as a percentage, taking the number of intracellular bacteria in control samples as 100%. Values are expressed as mean S.D. (error bars). A difference to the control was considered significant at the * *p* < 0.05 level. (**B**). The effect of pre-incubation of M-HeLa cells with inhibitor of EFGR (30 µM tyrphostin AG-1478) on the invasion intensity of *S. grimesii*. Control—intensity of invasion into untreated cells. The number of intracellular bacteria was estimated as a percentage, taking the number of intracellular bacteria in control samples as 100%. Values are expressed as mean S.D. (error bars). A difference to the control was considered significant at the * *p* < 0.05 level.

The accumulation of Ca^2+^ activates ADAM10 sheddase in eukaryotic cells. Therefore, we evaluated the contribution of ADAM10 to the intensity of *Serratia* invasion. A quantitative microbiological invasion assay showed that the maximum intensity of *Serratia* invasion was achieved when 1000 bacteria per cell were infected. The 10-fold increase and decrease in the number of *S. proteamaculans* cells during infection reduced the intensity of invasion by 4 times. The 10-fold increase and decrease in the number of *S. grimesii* during infection reduced the intensity of invasion by 10 and 2 times, respectively. To quantify the effect of inhibitors on the sensitivity of the cells to bacteria, the number of intracellular bacteria was estimated as a percentage, taking the number of intracellular bacteria after incubation with control cells as 100%. We showed that when infected with 1000 and 10,000 bacteria per cell, the inhibitor of ADAM10 metalloprotease reduces the intensity of *S. grimesii* invasion. When the M-HeLa cells were infected with 100 *S. grimesii* bacteria per cell or with 100–10,000 *S. proteamaculans* bacteria per cell, which did not lead to Ca^2+^ accumulation in the 2 h of incubation, the ADAM10 inhibitor did not affect the invasion (Figure 6A). Thus, the ADAM10 inhibitor inhibits *S. grimesii* invasion only when Ca^2+^ accumulates in host cells upon their infection with bacteria.

**Figure 7:**
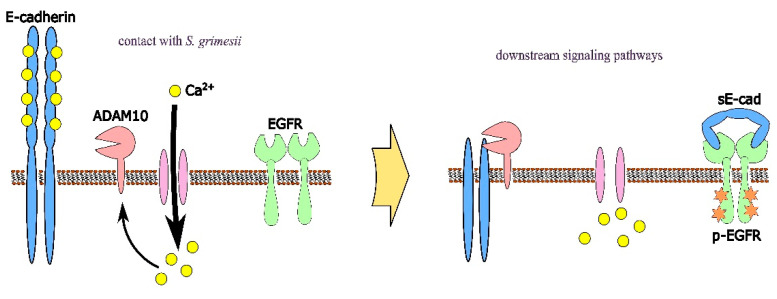
By inducing Ca^2+^ influx, *S. grimesii* infection may trigger ADAM10 activation, thereby leading to E-cadherin shedding. Cleavage of E-cadherin by sheddase results in the release of an extracellular soluble 80 kDa fragment of E-cadherin (sE-cad) from the cell surface [14]. The sE-cad can bind to EGFR and stimulate EGFR phosphorylation (p-EGFR) [19,20].

The catalytic activity of sheddases triggers the extracellular release of a soluble E-cadherin (sE-cad) fragment of about 80 kDa from the cell surface. The soluble E-cadherin can have a role in EGFR signaling independent of traditional EGFR ligands. It can bind to EGFR and stimulate EGFR phosphorylation, downstream signaling, and proliferation [19,20]. Previously, we have shown that the treatment of M-HeLa cells with the EGFR inhibitor reduced the invasion of *S. proteamaculans* [10] but increased the infection with *S. grimesii* at an MOI of 100 by 1.5 times [21]. To determine whether the EGFR is involved in *S. grimesii* invasion at the level of 1000 and 10000 bacteria per cell, we used the selective EGFR inhibitor tyrphostin AG-1478 (Figure 6B). With this number of bacteria per cell during infection, the EGF receptor was involved in *S. grimesii* invasion. In addition, the quantitative effect on the intensity of the bacterial invasion of ADAM10 inhibition was similar to the effect of the inhibition of signal transduction from EGFR. Thus, soluble E-cadherin may promote *S. grimesii* invasion by playing a role in EGFR signaling (Figure 7).

## 3. Discussion

In the body, cell adhesion plays an important role in various processes, from cell differentiation to proliferation and morphogenesis. [22]. Cell-to-cell adhesion is mediated by surface receptors, which determine not only intercellular adhesion but also the barrier properties of the epithelium and endothelium. These membrane-anchored receptors consist of surface-exposed and intracellular domains that are associated with components of the cytoskeleton [23]. Cytoskeletal rearrangements are necessary for bacterial invasion [24]. Therefore, adhesion receptors are often targeted by pathogens to take over and exploit the host mechanism for adhesion and entry into the eukaryotic cell [23]. E-cadherin is one such protein that pathogens target for entry and survival inside host cells [5]. Many pathogens target E-cadherin early in pathogenesis, such as the bacterial surface protein internalin A (InlA) of *Listeria monocytogenes* [3,5]. The interaction of InlA with E-cadherin initially results in the bacterial adhesion to the host cell surface and then, by manipulating the E-cadherin/β-catenin system, activates downstream signaling pathways that trigger *L. monocytogenes* endocytosis [4]. In addition, binding of the bacterial proteins FadA of *Fusobacterium nucleatum* and CagA of *Helicobacter pylori* to E-cadherin results to the disruption of E-cadherin/β-catenin complexes and causes the translocation of β-catenin to the nucleus, where β-catenin activates target genes [25,26].

This work aimed at studying the role of E-cadherin in the mechanism of *S. grimesii* and *S. proteamaculans* invasion. Using real-time RT-PCR, it was shown that the E-cadherin gene expression increased in response to infection with *Serratia*. Confocal microscopy made it possible to evaluate the redistribution of E-cadherin as a result of incubation of Caco-2 cells with bacteria. It was shown that this receptor accumulates along the host cell perimeter in response to *S. grimesii* infection and in granules in response to *S. proteamaculans* infection. However, the confocal microscopy did not allow us to determine the distribution of E-cadherin in the M-HeLa cells. According to the Western blot analysis, E-cadherin was detected in the Caco-2 cells in a full-length form and in the M-HeLa cells in a truncated form only in response to incubation with bacteria. Transfection with siRNA targeting E-cadherin inhibited *S. proteamaculans* invasion into the Caco-2 cells by 35% but did not inhibit the invasion in M-HeLa cells. On the other hand, transfection with siRNA targeting E-cadherin inhibited *S. grimesii* invasion into both cell lines by 20%. Thus, full-length E-cadherin is involved in the invasion of *S. proteamaculans* more than in the invasion of *S. grimesii*, and truncated E-cadherin can only promote the invasion of *S. grimesii*.

Cleavage of intercellular junction proteins by secreted bacterial proteases is one of the mechanisms that facilitate bacterial entry into host cells. The serine protease HtrA of *H. pylori*, enteropathogenic *Escherichia coli*, *Shigella flexneri*, and *Campylobacter jejuni*, which is important for bacterial protein folding, promotes the bacterial entry by specifically cleaving cadherin, which leads to the destruction of the epithelial barrier [27]. In addition, bacteria can recruit host proteases such as ADAMs and MMPs (matrix metalloproteinase). ADAMs, a family of zinc-dependent transmembrane metalloproteases, are involved in the extracellular domain shedding of various membrane proteins. Sheddase ADAM10 cleaves E-cadherin, which affects the cell-to-cell adhesion, cell migration, and proliferation of epithelial cells [14]. Full-length E-cadherin is cleaved by metalloprotease near the transmembrane domain to form the 80 kDa N-terminal fragment (soluble E-cadherin) and the 38 kDa C-terminal fragment (CTF1), which can be further processed by γ-secretase-like activity into a 33 kDa soluble fragment (CTF2) [14]. ADAM10 is activated by Ca^2+^ influx in the host cells in particular due to the formation of pores in the cytoplasmic membrane [14]. The pore-forming delta toxin of *Clostridium perfringens* and alpha toxin of *Staphylococcus aureus* are capable of reducing the cell surface accumulation of E-cadherin by enhancing ADAM10 sheddase activity [28,29]. Similarly, the ShlA of *S. marcescens* and pore-forming toxin exolysin A (ExlA) of *Pseudomonas aeruginosa* can disrupt intercellular contacts of epithelial cells as a result of E-cadherin cleavage by Ca^2+^-activated ADAM10 [15].

Previously, we showed that *S. proteamaculans* bacteria synthesize the active ShlA toxin, while *S. grimesii* bacteria do not synthesize this toxin [16]. However, this work showed that, after 2 h of incubation with the bacteria, Ca^2+^ only accumulated in the cells infected with *S. grimesii*. The activation of the ShlA toxin reduced Ca^2+^ accumulation in the host cells upon infection with *Serratia*. Thus, *S. grimesii* and *S. proteamaculans* cause the ShlA-independent accumulation of Ca^2+^ in host cells, which can promote invasion. The increase in intracellular Ca^2+^ links receptor activation to downstream signaling pathways. Bacteria can induce an increase in free cytosolic Ca^2+^ in host cells, which is involved in signal transduction at various steps of bacterial infection [30]. During the infection of host cells with bacterial pathogens, among the wide variety of processes in which Ca^2+^ signaling is involved, the control of cytoskeletal reorganization and the expression of genes that lead to the accumulation and secretion of pro-inflammatory mediators is of particular importance [30]. The *L. monocytogenes* exotoxin (listeriolysin O) forms large pores across the host cell plasma membrane, causing a rapid influx of extracellular Ca^2+^. The Ca^2+^ influx leads to the activation of the small Rho-GTPase Rac1, which mediates the de novo assembly of the actin cytoskeleton required for the *L. monocytogenes* internalization [31].

*Serratia*-infection-induced Ca^2+^ influx can trigger ADAM10 activation, thereby leading to E-cadherin shedding. The cleavage of E-cadherin by sheddase results in the release of an extracellular soluble 80 kDa fragment of E-cadherin (sE-cad) from the cell surface. The sE-cad can bind to the surface of other cells with full-length E-cadherin, changing the cadherin-dependent cellular behavior [20]. It can have also a role in EGFR signaling independent of traditional EGFR ligands. The sE-cad can then bind to EGFR and stimulate EGFR phosphorylation, downstream signaling, and proliferation [19,20]. We showed that EGFR and sheddase ADAM10 are involved in *S. grimesii* invasion at an MOI of 1000 and 10,000. An addition, the quantitative effect on the intensity of bacterial invasion of ADAM10 inhibition is similar to the effect of the inhibition of signal transduction from EGFR. Apparently, as a result of infection with *S. grimesii*, Ca^2+^ accumulates in the host cells and may activate ADAM10 sheddase, which can promote invasion by cleaving E-cadherin and, as a result, triggering EGFR signaling. Thus, the invasion of *S. proteamaculans* can only be promoted by full-length E-cadherin, and the invasion of *S. grimesii* can be promoted by both full-length and truncated E-cadherin.

## 4. Materials and Methods

### 4.1. Cell Cultures, Bacterial Strains, and Growth Conditions

The cervical carcinoma M-HeLa, adenocarcinoma alveolar basal epithelial A549, and colorectal adenocarcinoma Caco-2 cell lines were obtained from the “Vertebrate cell culture collection” (Institute of Cytology, St. Petersburg, Russia) supported by the Ministry of Science and Higher Education of the Russian Federation (agreement ›075-15-2021-683). The M-HeLa cells were grown in MEM medium containing 1% nonessential amino acids (NEAAs) (Sigma-Aldrich, Munich, Germany) and 10% fetal bovine serum (Sigma-Aldrich, Munich, Germany). The A549 and Caco-2 cells were grown in DMEM medium contained 10% fetal bovine serum (Sigma-Aldrich, Munich, Germany).

*S. proteamaculans* strain 94 was isolated as described earlier [32]. *S. grimesii* strain 30063 was obtained from the German Collection of Microorganisms and Cell Cultures (DSMZ). *S. proteamaculans* and *S. grimesii* were grown as previously described [21].

### 4.2. siRNA Transfection

The expression of host cell proteins was inhibited using siRNA targeting E-cadherin (sc-35242) (Santa Cruz, Dallas, TX, USA). Transfection of siRNAs was performed using siRNA Transfection Reagent (sc-29528) as recommended by the manufacturer (Santa Cruz, Dallas, TX, USA). The RNA interference efficiency was controlled by Western blotting.

### 4.3. Quantitative Invasion Assay

Efficiency of invasion was evaluated by a quantitative invasion assay [33,34]. Cells forming a 50–70% monolayer were transfected with RNA or treated with inhibitors in DMEM for 30 min before adding bacteria. To inhibit the Rho/ROCK pathway, the cells were pretreated with 10 μM Y-27632 (Sigma-Aldrich, Munich, Germany). To inhibit the ADAM10 metalloprotease, the cells were pretreated with 20 μM GI 254023X (Santa Cruz, Dallas, TX, USA). To inhibit the EGFR, the cells were pretreated with 30 mkM tyrphostin AG-1478 (Sigma-Aldrich, Munich, Germany) for 1 h before adding bacteria. The host cells were infected at a ratio of 100, 1000, or 10,000 bacteria per cell, and the intensity of invasion was determined as previously described [21]. After co-cultivating the host cells and bacteria, the proportions of viable cells were measured after staining with trypan blue by counting in a Goryaev chamber. Cell viability in all experiments was at least 90%. The intensity of invasion was determined as the number of CFU normalized to the number of viable eukaryotic cells determined after incubation with bacteria. The results for each experiment were the average of an assay performed in triplicate and were independently repeated three times.

### 4.4. Western Blot Analysis

After incubation with *Serratia*, the amounts of the proteins in the cells were analyzed using Western blot analysis as described previously [21]. Antibodies against E-cadherin (ab15148) at a dilution of 1:500 (Abcam, Cambridge, UK) were used to stain the E-cadherin.

### 4.5. Fluorescence Microscopy

Cells were grown at 37 °C in an atmosphere of 5% CO_2_ on coverslips until a 70–80% monolayer was formed. If necessary, before the experiment, the cells were incubated with a selective inhibitor, i.e., 20 μM ADAM10 metalloprotease (GI 254023X), for 30 min. The bacteria *S. grimesii* and *S. proteamaculans* were grown in LB medium (Sigma-Aldrich, Munich, Germany) with aeration for 24 h at 37 °C and 30 °C, respectively. The bacterial suspension was centrifuged at 9600 g for 8 min. The pellet was resuspended in DMEM medium and added to the eukaryotic cells at a ratio of 10,000 bacteria per cell. The bacteria were deposited on the surface of the host cell by centrifugation for 5 min at 2000 rpm. The host cells and bacteria were co-cultivated at 37 °C in 5% CO_2_ for 2 h. The samples were stained and analyzed as previously described [10]. Antibodies against E-cadherin (ab15148) at a dilution of 1:100 (Abcam, Cambridge, UK) were used to stain the E-cadherin.

### 4.6. Real-Time RT-PCR

Total RNA was extracted from the cells using a Dia-M Extraction Kit according to the manufacturer’s instructions (Dia-M, Moscow, Russia). Gene expression was assessed using real-time PCR as described previously [21]. The gene-specific primer pairs (Evrogen, Moscow, Russia) designed using the BLAST-primer software and used for real-time PCR are listed in Table 1. Qualitative analysis prior to the real-time PCR confirmed that only one fragment with a molecular weight calculated using the BLAST software was synthesized from the cDNA template with the gene-specific primer pairs.

### 4.7. Quantitative Imaging Cytometry for Ca^2+^ Accumulation in Cells Analysis

The membranes of the eukaryotic cells were stained with a PKH26 red fluorescent fell linker kit (Sigma-Aldrich, Munich, Germany) according to the manufacturer’s instructions. After 2 washes with PBS, 5 × 10^6^ M-HeLa cells were incubated with 1 mL of PKH26 for 5 min in the dark. The dye was inactivated by adding fetal bovine serum for 1 min. After washing with PBS, 25 × 10^4^ cells were seeded per well in a 12-well plate. Cells were incubated for 2.5 h in a CO_2_ incubator to attach them to the plate. Ca^2+^ was stained with Fluo-4, AM (Invitrogen, Waltham, MA, USA), according to the manufacturer’s protocol. After 1 h of incubation in DMEM medium with the Fluo-4, the medium was changed to DMEM with 0.5 µg/mL Hoechst 33342 (Invitrogen, Waltham, MA, USA) for 30 min to stain the DNA. Either 500 or 50 bacteria per cell were added to the stained cells in fresh DMEM and pelleted by centrifugation for 5 min at 2000 rpm. For this, *S. grimesii* were grown in LB medium for 24 h at 37 °C and *S. proteamaculans* were grown in LB medium for 48 h at 30 °C in the absence or presence of 0.3 mM 2,2′-bipyridyl. Ca^2+^ accumulation in the host calls was analyzed by frame-by-frame imaging using a CQ1 confocal imaging cytometer (Yokogawa, Japan). Cell images were taken every 5 min for an 18 h period using a system of lasers with wavelengths of 405 (blue fluorescence), 488 (green fluorescence), and 561 nm (red fluorescence). The images were analyzed using the CQ1 software. Cells were identified by Hoechst 33342-stained nuclei, and their perimeter was determined by PKH26-stained membranes. Then, the mean intensity of the Fluo-4-stained Ca^2+^ per cell was determined in the obtained images.

### 4.8. Statistical Analysis

Each quantitative experiment was repeated at least three times. Significance testing in the comparisons was based on Student’s *t*-tests for pairs and analysis of variance (ANOVA) with the Excel Data Analysis Pack. A difference was considered significant at the *p* < 0.05 level.

## Figures and Tables

**Table 1:** Gene-specific primer pairs.

Target Gene	Primer Sequences
E-cadherin	Forward 5′-ATGCTGATGCCCCCAATACC-3′
	Reverse 5′-GGGGGCTTCATTCACATCCA-3′
β-actin	Forward 5′-AATCTGGCACCACACCTTCTACA-3′
	Reverse 5′-GACGTAGCACAGCTTCTCGTTA-3′
GADPH	Forward 5′-GGCATGGACTGTGGTCATGAG-3′
	Reverse 5′-TGCACCACCAACTGCTTAGC-3′

## Data Availability

Data available on request from the author.
